# Role of reactive oxygen species and mitochondrial damage in rheumatoid arthritis and targeted drugs

**DOI:** 10.3389/fimmu.2023.1107670

**Published:** 2023-02-09

**Authors:** Weiyao Jing, Cui Liu, Chenghong Su, Limei Liu, Ping Chen, Xiangjun Li, Xinghua Zhang, Bo Yuan, Haidong Wang, Xiaozheng Du

**Affiliations:** ^1^ Department of Acupuncture-Moxibustion and Tuina, Gansu University of Chinese Medicine, Lanzhou, China; ^2^ Department of Rheumatic and Bone Disease, Gansu Provincial Hospital of Traditional Chinese Medicine, Lanzhou, China; ^3^ Department of Acupuncture, Gansu Provincial Hospital of Traditional Chinese Medicine, Lanzhou, China; ^4^ Department of Acupuncture and Pain, Affiliated Hospital of Gansu University of Traditional Chinese Medicine, Lanzhou, China

**Keywords:** rheumatoid arthritis, reactive oxygen species, mitochondrial damage, targeted drugs, oxidative stress

## Abstract

Rheumatoid arthritis (RA) is an autoimmune disease characterized by synovial inflammation, pannus formation, and bone and cartilage damage. It has a high disability rate. The hypoxic microenvironment of RA joints can cause reactive oxygen species (ROS) accumulation and mitochondrial damage, which not only affect the metabolic processes of immune cells and pathological changes in fibroblastic synovial cells but also upregulate the expression of several inflammatory pathways, ultimately promoting inflammation. Additionally, ROS and mitochondrial damage are involved in angiogenesis and bone destruction, thereby accelerating RA progression. In this review, we highlighted the effects of ROS accumulation and mitochondrial damage on inflammatory response, angiogenesis, bone and cartilage damage in RA. Additionally, we summarized therapies that target ROS or mitochondria to relieve RA symptoms and discuss the gaps in research and existing controversies, hoping to provide new ideas for research in this area and insights for targeted drug development in RA.

## 1 Introduction

Rheumatoid arthritis (RA) is an autoimmune disease characterized by synovial inflammation and pannus, bone, and cartilage damage. It has a global prevalence of approximately 0.5–1% and occurs more commonly in women than in men (1). Genetics is a key factor in the development of RA, and sex, smoking, and environmental factors influence the development of RA (2). In RA, permanent T-cell and monocyte-mediated synovial inflammation are the underlying cause of disease progression. Pannus is a characteristic pathological product of RA, with tumor-like properties that drive synovial proliferation and bone erosion, which can eventually lead to disability and seriously affect patients’ quality of life (3). The onset of RA involves not only the joints but also cardiovascular disease and interstitial lung disease, which are serious complications that result in a shorter life expectancy in patients with RA (4, 5). Mitochondria are an important source of reactive oxygen species (ROS) within most mammalian cells. However, ROS accumulation may activate mitochondrial permeability transition pore (mPTP) and inner membrane anion channel (IMAC) opening. Longer mPTP openings may release a burst of ROS, which contributes to mitochondrial damage (6). ROS and mitochondrial damage are inextricably linked to several key pathological processes in RA (7–10), and the regulation of mitochondrial function, clearance of ROS, and alleviation of oxidative stress are currently popular targets for RA treatment (11, 12). In this review, we describe the role of ROS and mitochondrial damage in the major pathological changes that occur in RA, summarize the drugs targeting ROS or mitochondria for RA treatment, and suggest their relatively weakly studied but value-rich directions in RA.

## 2 ROS and mitochondrial damage in RA

Mitochondria are organelles with a bilayer membrane structure that supply the organism with adenosine triphosphate mainly through the oxidative phosphorylation process. This process comprises five mitochondrial respiratory chain enzyme complexes; complexes I–IV constitute the electron transport chain, and complex V is ATP synthase (13). The electron transport chain oxidizes nicotinamide adenine dinucleotide and flavin adenine dinucleotide produced by glycolysis and the tricarboxylic acid (TCA) cycle and pumps protons out of the mitochondrial inner membrane to produce a proton gradient. Thus, mitochondria can transfer electrons and regulate the body’s oxidation/reduction (redox) reactions (14). ROS mainly include free radicals, such as superoxide anions ( ${\text{O}}_{2}^{-}$ ) and hydroxyl radicals (OH^-^), and non-radical oxidants, such as hydrogen peroxide (H_2_O_2_) and singlet oxygen (^1^O_2_) (15). ROS are mainly derived from the process of electron transfer from the mitochondrial electron transport chain complex to O_2_, with complexes I and II producing ${\text{O}}_{2}^{-}$ in the mitochondrial matrix and complex III producing ${\text{O}}_{2}^{-}$ in the matrix and membrane interstitium, which is the precursor of most ROS. ${\text{O}}_{2}^{-}$ in the mitochondrial matrix is converted to H_2_O_2_ by manganese superoxide dismutase, and copper- and zinc-containing superoxide dismutase mainly converts ${\text{O}}_{2}^{-}$ in the membrane interstitium and cytoplasm, which is eventually catabolized to H_2_O. Under normal conditions, ROS production and elimination are balanced and play a role in promoting immunity and regulating the cell cycle (16).

**Figure 1:**
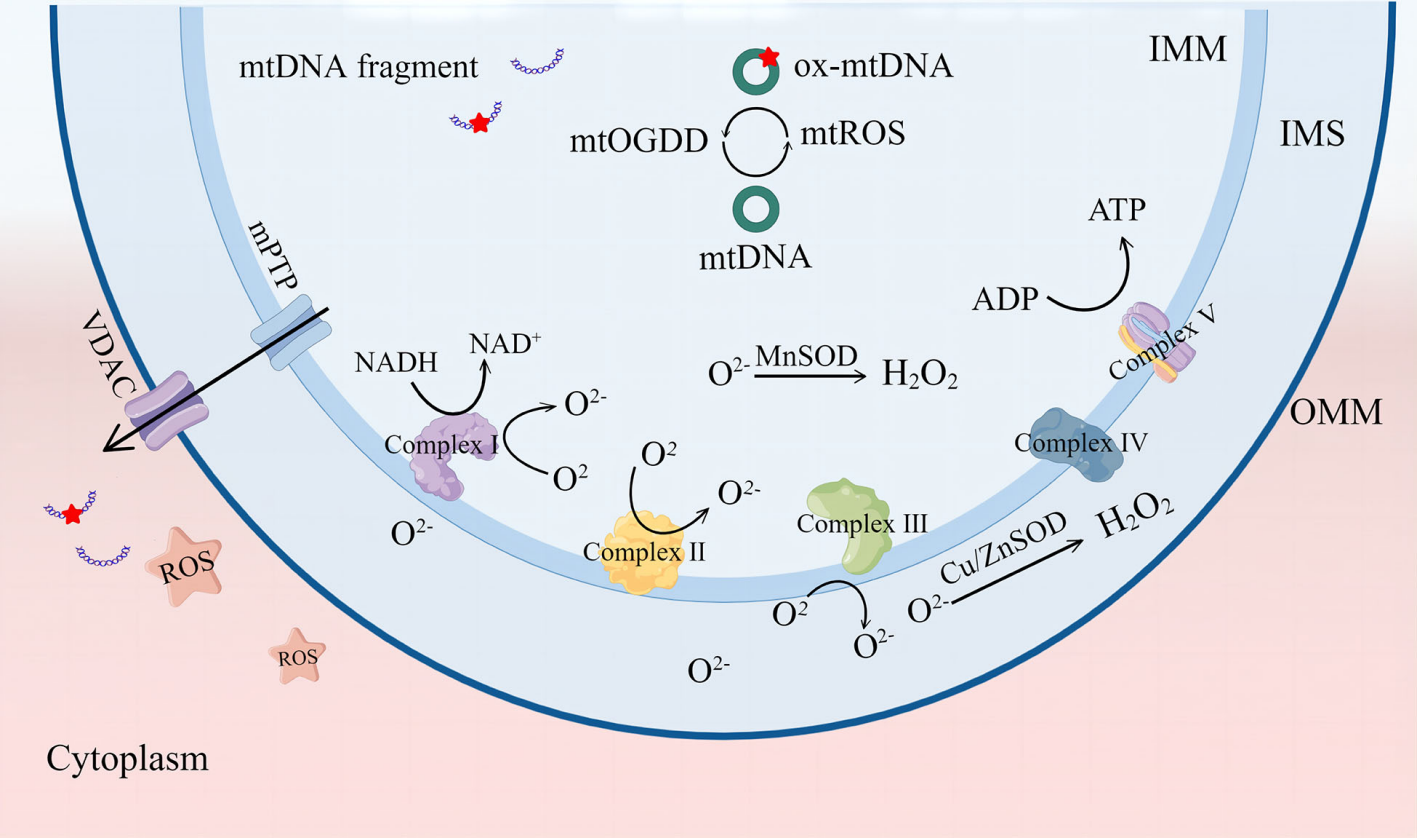
The mitochondrial respiratory chain complex is the main site for ROS production. ROS can be catalyzed as H_2_O_2_ by MnSOD or Cu/ZnSOD, and ROS accumulation causes mtDNA damage and leakage into the cytoplasm through mPTP and VDAC channels. VDAC, voltage-dependent anion channel. mPTP, mitochondrial permeability transition pore. ROS, reactive oxygen species. SOD, superoxide dismutase; IMM, inner mitochondrial membrane; IMS, intermembrane space; OMM, mitochondrial outer membrane. By Figdraw.

Redox homeostasis is determined by the balance between ROS generation and ROS quenching capacity. When the equilibrium is tilted toward ROS production, conditions are created for oxidative stress. Mild ROS cumulation can cause oxidation of essential mitochondrial components; in extreme cases, it can irreversibly cause mitochondrial damage. Cardiolipin (CL) is a dimeric phospholipid with a high content of unsaturated fatty acids mainly distributed in the inner mitochondrial membrane. CL is particularly prone to ROS-induced oxidative attacks. Oxidized CL redistributes from the inner mitochondrial membrane to the outer membrane (17, 18). The accumulation of oxidized CL on the OMM results in mPTP formation. These mPTP changes result in more ROS formation, known as “ROS-induced ROS release” (RIRR), and cause an oxidative stress response (6). In fact, mitochondria-derived ROS can self-destruct mitochondria. ROS in the matrix can cause oxidative damage to mtDNA, leading to mutation of mtDNA, inhibition of mitochondrial aerobic respiration, reduction of ATP production, and disruption of the mitochondrial membrane potential (ΔΨm). These changes eventually lead to mitochondrial depolarization. Recent studies have shown that persistent deletion of mtDNA leads to irreversible mitochondrial damage (19). Studies have shown that glutathione (GSH) peroxidase 4 inhibitor (RSL3) induces high ROS expression in mouse embryonic fibroblasts, enhanced mitochondrial fragmentation, mitochondrial membrane potential loss, reduced mitochondrial respiration, and ROS scavenger mitoquinone (MitoQ)-preserved mitochondrial integrity and function (20) (**Figure 1**).

In patients with RA, tPO_2_ in the joint cavity is significantly lower than that in normal tissues (21), and joint cavity hypoxia is the underlying condition for ROS accumulation and mitochondrial damage in synovial tissue (22). ROS have a strong correlation with the level of disease activity in patients with RA. They are positively correlated with C-reactive protein (CRP) and anti-cyclic peptide-containing citrulline levels in patients’ blood and can be used as an indirect assessment indicator of the degree of synovial inflammation in patients with RA (23–26). Previous studies have reported that 18 mitochondria-related proteins were upregulated and four proteins were downregulated in patients with RA, with significant mtDNA damage and reduced mitochondrial membrane potential, superoxide, and cellular ATP levels. These findings indicate that mitochondrial damage plays an important role in RA (27–29). In fact, ROS can modulate immune cell function and activation processes (30, 31). Mitochondria affect their cell cycle and inflammation tendency by adjusting metabolism or cell death and participate in RA inflammation (32, 33). Fibroblast-like synoviocytes (FLS) are the key effector cells in RA inflammation. ROS and mitochondrial damage regulate FLS proliferation, invasion, and production of inflammatory factors and can affect FLS survival by affecting apoptosis, autophagy, and other processes mediated by FLS (34). ROS can cause mtDNA accumulation in the cytoplasm and in turn act as pathogen-associated molecular patterns (PAMPs) to activate multiple inflammatory pathways and mediate inflammatory responses (19). In synovial tissue, ROS promote pannus formation by inducing angiogenesis and angiogenesis by upregulating HIF, VEGF, and Notch expression (35–37). Changes in mitochondrial membrane proteins and abnormal mitochondrial respiration affect this process (38). Besides, due to the different responsiveness of osteoclasts and osteoblasts to ROS, ROS induce the differentiation and activation of osteoclasts and mitochondrial damage and apoptosis of osteoblasts. This favors the balance toward the process of bone resorption, causing bone destruction, while ROS affect the release of MMPs and activity of chondrocytes, exacerbating cartilage damage (39–41). Therefore, both ROS and mitochondrial damage are involved in the development of RA (42, 43). In a randomized controlled trial, Yoga was shown to enhance mitochondrial quality, reduce oxidative stress marker production, and improve the Disease Activity Score-Erythrocyte Sedimentation Rate and Health Assessment Questionnaire-Disability Index scores in patients with RA, which may be a beneficial adjunct to training for RA (44).

## 3 Role of ROS and mitochondrial damage in RA inflammation

Synovial inflammatory response is the central mechanism of RA lesions and main factor leading to pannus (45, 46) and cartilage destruction (47, 48). The subsynovial and lining layers are altered in patients with RA, and T-cells, B cells, and dendritic cells are widely distributed in the subsynovial region (49). They drive synovial inflammation together (50, 51). The synovial lining in RA is dominated by synovial macrophages and FLSs, which are highly activated and produce a large number of pro-inflammatory factors, chemokines, and growth factors (52). These factors can activate FLSs and mediate proliferation, anti-apoptosis, erosion, migration, and other pathological behaviors (53, 54). Mitochondrial damage and oxidative stress can affect immune cell metabolic processes and promote inflammatory behavior (55, 56). Additionally, ROS and the products of mitochondrial damage are good activators of inflammatory response.

### 3.1 Regulation of immune cell function

#### 3.1.1 T cell

In RA, immune cells function abnormally, producing a large number of inflammatory factors, such as tumor necrosis factor (TNF)-α, interleukin (IL)-6, and IL-17, which are distributed in clusters in the diseased joints, causing an inflammatory storm (57–59). Mitochondria-derived ROS assist in antigen presentation and are important regulators of the T-cell cycle and function (30, 60). Studies have shown that ROS promote Th17 differentiation and increase IL-17 production (61). The use of ROS scavengers significantly inhibits Th17 differentiation (62). The *IEX-1* gene plays a key role in this process, and *IEX-1* overexpression significantly inhibits mtROS production. Collagen-induced arthritis (CIA) mice with knocked-out *IEX-1* have higher amounts of Th17 and exhibit more severe joint inflammation (63). Additionally, hypoxia not only induces ROS accumulation but also induces high expression of HIF-1α (64). Hyperbaric oxygen therapy reduces the levels of IL-17a, CRP, and rheumatoid factor in CIA mice by regulating the expression of HIF-1α and promotes the differentiation of Tregs, alleviating oxidative stress and inflammatory response (65). CD3^+^ T-cells and CD68 macrophages cultured in a hypoxic environment exhibit a stronger inflammatory response (21), confirming the critical role of hypoxia in immune inflammation in RA (27). The hypoxic environment in the RA joint cavity leads to altered immune cell metabolism, impaired oxidative phosphorylation processes, and significantly increased aerobic glycolytic activity in organisms with RA (66–68). This process causes lactate accumulation and promotes the inflammatory behavior of immune cells (69, 70). T-cells in RA prefer the glycolytic pathway to break down glucose into ATP; however, the underlying mechanism remains unknown, and its relationship with ROS is not yet clear (71, 72). Indeed, the role of ROS in T-cells remains paradoxical, as ROS can activate the expression of nuclear factor of activated T-cells (NFAT) and induce c-MYC transcription, contributing to T-cell activation (73). The accumulation of ROS leads to the upregulation of GSH expression due to the presence of an oxidative coordination system. GSH can inhibit NFAT and MYC activation, whereas GSH production protects the integrity of T-cell metabolism (74).

In RA, reduced mitochondrial DNA biostability and mtDNA leakage into the cytoplasm due to the lack of DNA repair nuclease (MRE11A) increase the pro-inflammatory tendency of T-cells, whereas upregulation of MRE11A expression reduces mitochondrial damage and has an inhibitory effect on T-cell scorching and immune inflammation (32, 75). The expression of malondialdehyde (MDA) H_2_O_2_ was significantly increased in the paw tissue of CIA mice, and intervention with curarelinone, the active ingredient of bitter ginseng, significantly inhibited oxidative damage and decreased the phosphorylation levels of signal transducer and activator of transcription (STAT)1, STAT3, and ratio of Th1 and Th17 cells in lymph nodes (62). Yun et al. (76) used rosmarinic acid intervention to induce cytochrome C release from the mitochondria and induce apoptosis in activated T-cell subsets in patients with RA by blocking mitochondrial depolarization. Mesenchymal stem cells (MSCs) have potential in RA treatment (77, 78). Th17 cells co-cultured with bone marrow-derived MSCs (BM-MSCs) could reduce TNF-α and IL-17 production and restore T-cell oxidative phosphorylation activity in a contact-dependent manner through a mechanism related to mitochondrial transfer. Th17 cells could reduce IL-17 production through the uptake of healthy mitochondria in BM-MSCs with immunomodulatory effects. RA-synovium-derived MSCs have impaired mitochondrial transfer to Th17 cells, which may be a key reason for the persistent inflammatory response in RA synovial tissues (79, 80). Furthermore, mitochondrial transfer treatment corrects cellular metabolic defects, increases ATP production, and decreases ROS levels. Therefore, mitochondrial transfer has therapeutic potential in regulating immune cell function in RA (81–83).

#### 3.1.2 B cell

B cells play an important role in the immune response to RA. On the one hand, B cells can produce autoantibodies, such as rheumatoid factor (rheumatoid factor, RF) and anti-citrulline protein antibody (ACPA), which can form immune complexes that are deposited in joints, and promote the inflammatory process through complement and cell activation. On the other hand, B cells, as potent antigen presenting cells (APC), activate T cells through the expression of co-stimulatory molecules (84). B cell depletion therapy highlights some advantages in RA therapy. Rituximab (RTX) is a human-mouse chimeric monoclonal antibody targeting the B cell-specific antigen, CD20, that induces B cell death through antibody-dependent cytotoxicity and phagocytosis mediated by Fc receptor γ. In anti-TNF inadequate responder patients with RA, RTX can reduce the levels of ESR, CRP, and RF, and improve clinical symptoms (85). However, the therapeutic effect of RTX is seemingly limited by the number of B cells in the synovial tissue.

BCR signaling is a critical step in controlling B cell maturation and differentiation. Endogenous ROS can regulate the level of BCR signaling through a reversible inhibition of protein tyrosine phosphatase activity. This process is associated with a reduced activation threshold of the spleen tyrosine kinase (SYK) (86), removing ROS by a scavenger, N-acetylcysteine, and resulting in impaired BCR-induced activation (87). ROS affects the proliferation of B cells and participates in the CD47-mediated G1 phase arrest of B cells, and clearing ROS can effectively eliminate this response (31). Additionally, the fate of B cells is greatly correlated with mitochondrial function. According to studies, mitochondrial mass and membrane potential were significantly higher in B cells of class-switch recombination (CSR) type, whereas B cells of plasma cell differentiation (PCD) type showed a decrease in mitochondrial mass and membrane potential (33). New studies have revealed that mitochondrial fission factor can specifically bind to TRAF 3 to regulate the progression of B cell apoptosis (88). These studies confirmed the association between ROS and mitochondria and B cells. In a mouse model of RA, ROS mediated the tolerance of B cells to autoantigens, and the mutated NCF 1 gene caused ROS deficiency, disrupting resistance to arthritis (89).

#### 3.1.3 Neutrophil

Neutrophils represent about 60% of the total leukocytes. They are the first cells to migrate to the site of inflammation and infection. Neutrophils exhibit multifunctional heterogeneity in orchestrating adaptive immune responses. Through exudation, neutrophils migrate from the bloodstream to the involved tissues and release degrading enzymes and ROS to play a cytotoxic role during infection (90). Evidence suggests that neutrophil extracellular trap (NET), peptidylarginine deiminase (PAD) activation, and citrullinated peptide generation are the crux of RA pathogenesis. Activated neutrophils release PAD enzymes that promote citrullination of synovial tissue. Recognition of a citrullinated peptide by MHC II promotes T cell activation and autoantibody production. Neutrophils accumulate heavily in RA synovial fluid and synovial tissue, and RA-FLS have the ability to internalize NET-associated citrullinated peptides, acquire antigen-presenting cells (APC), and present them to CD4+ T cells to induce an autoimmune response (91).

The level of neutrophil ROS in the synovial tissue of patients with RA is significantly higher than that in patients with other forms of arthritis. The ROS produced by neutrophil degranulation may affect the degree of oxidative stress in RA (92). A clinical study observed an increase in the intracellular and mitochondrial oxidative stress and decreased antioxidant enzymes. The intracellular level of ROS in polymorphonuclear neutrophils (PMNs) were positively correlated with inflammatory response and disease severity (93). Furthermore, RA synovial tissue highly expressed neutrophil chemokines and ROS. They lost their migratory properties and remained resident in joints to cause inflammation and bone destruction by recruiting and activating immune cells (94). In RA, neutrophils form a vicious cycle with oxidative stress and inflammation. The proinflammatory microenvironment in RA synovial tissue combined with high concentrations of ROS has been shown to jointly induce neutrophils to neutrophil-dendritic (N-DC) differentiation and show more ROS generation and inflammation tendency (95). Methotrexate reduces the expression of ROS, CD177, and CD11b in circulating neutrophils of patients with RA, which may be one of the mechanisms underlying its treatment of RA (96). mitochondrial formyl peptides (mtNFPs) is one of the key molecular patterns associated with mitochondrial damage, with increased circulating mtNFPs in RA patients, associated with disease activity.mtNFPs Neutrophil activation can be induced *via* formyl peptide receptor 1 (FPR 1) (97).Plastoquinonyl-Decyl-triphenylphosphonium bromide (SkQ1), an antioxidant targeting mitochondria, continuously removes ROS from mitochondria and protects cardiolipin on the inner mitochondrial membrane from oxidisal damage. SkQ1 intervention improved arthritis index and pathological injury severity in RA rats and promoted apoptosis of neutrophils *in vitro*. This mechanism may be one of the mechanisms by which SkQ1 exerts its pharmacological activity (98).

### 3.2 Modulation of synovial pathological behavior

RA-FLSs have a distinctly aggressive nature; its cause is related to synovial inflammatory stimulation and epigenetic modifications (99, 100). The hypoxic environment of synovial tissue and mitochondrial damage are responsible for synovial inflammation and oxidative DNA damage, which increase the aggressiveness of FLSs (101). Microsatellite instability was reportedly significantly higher in RA synovial tissues than in osteoarthritis (OA) synovial tissues, indicating a decreased DNA mismatch repair (MMR) capacity and severe DNA damage. Oxidative stress can downregulate the DNA MMR system in RA-FLSs by inhibiting *hMSH6*. The oxidative stress environment can interfere with the repair process of single-base mutations and DNA damage by inhibiting *hMSH6* (102, 103), and mutations in genes, such as *P53* and *LBH*, can lead to pathological behaviors, such as invasion and proliferation of RA-FLSs (104, 105). ROS accumulation leads to metabolic abnormalities in RA-FLSs, with decreased mitochondrial oxidative phosphorylation and ATP reserve capacity but increased glycolytic activity in RA-FLSs, promoting RA synovial inflammation (106). High expression of HIF-2α in RA joints induces the secretion of multiple chemokines, promotes FLS migration and invasion, induces pannus formation, and aggravates bone destruction (107, 108). Additionally, ROS and HIF-2α can enhance the migration of RA-FLSs by regulating CD70 expression, whereas reduced oxidative damage can inhibit its migration (109). The level of mtROS in Treg cells of patients with RA increases with disease activity, and peripheral blood mononuclear cells (PBMCs) cultured with ROS inhibitors significantly reduce RA-FLS inflammation (110). This appears to be a possible reason for the ozone treatment of RA (111).

ROS accumulation leads to the corresponding activation of the Keap1/Nrf2 pathway, and Nrf2 transcribes various antioxidant enzymes, including superoxide dismutase (SOD), heme oxygenase-1 (HO-1), and GSH. It is a key pathway in the fight against oxidative damage (112). Knockdown of Nrf2 leads to RA-FLS activation and promotes its proliferation (113). The intervention of RA-FLSs with resveratrol significantly increases the expression of Nrf2 and HO-1, reduces the production of ROS and MDA and activation of nuclear factor kappa-B (NF-κB) p65, inhibits the proliferation and migration of RA-FLSs, and promotes its apoptosis (114, 115). Similarly, mitochondrial damage plays a key role in the pathological behavior of FLSs, and studies have shown that healthy mitochondrial transfer inhibits LPS-induced FLS proliferation and migration and promotes apoptosis, which can reduce the inflammatory response (80). In contrast, inducing mitochondrial damage in normal FLSs can promote NF-κB pathway activation and ROS production and increase the secretion of inflammatory factors (116). Adenosine 5’-monophosphate (AMP)-activated protein kinase (AMPK) is a key regulatory protein of mitochondrial mass (117), and its activation significantly inhibits the activation and proliferation of RA-FLSs (118). In fact, the high expression of glycogen synthase-1 in RA-FLSs can lead to the excessive accumulation of glycogen and inhibit AMPK expression, leading to the high expression of matrix metallopeptidase (MMP)-1, MMP-9, IL-6, and CCL-2, along with increased proliferation and migration of FLSs. Intervention with the AMPK-specific agonist, AICAR, blocked RA-FLS activity and improved symptoms in CIA rats (119). AMPK can remove damaged mitochondria by inducing mitochondrial autophagy and regulating mitochondrial quality, which reduces ROS production while removing damaged mitochondria. However, the effect of mitochondrial autophagy has two sides (120, 121). On the one hand, promoting mitophagy can inhibit multiple pathological behaviors of FLSs and reduce the inflammatory response induced by mtDNA and ROS (122–124). On the other hand, mitochondrial autophagy, as a means for cells to deal with stress, may contribute to cell survival. Inhibition of mitochondrial autophagy has been proposed to have the ability to contribute to FLS apoptosis, and the effects mediated by the different periods of mitochondrial autophagy vary (125–127). Therefore, the role and mechanisms underlying mitochondrial autophagy in RA-FLSs and RA need to be explored further.

Lipid peroxidation caused by ROS is involved in apoptosis, autophagy, and ferroptosis (128). However, it remains controversial in regulating RA-FLS cell death. H_2_O_2_ reduced mitochondrial membrane potential and increased ROS production in treated RA-FLS and activation of caspase-3, caspase-9, and Bax to induce FLS apoptosis, a process associated with oxidative stress-mediated activation of macrophage stimulating 1 (Mst1) and inhibition of the AMPK-Sirt1 signaling pathway (129). Exposure to Mitomycin C (MMC) has been shown to increase ROS production in RA-FLS and disrupt ΔΨ m, increasing the release of mitochondrial cytochrome c and the ratio of Bax/Bcl-2 and inducing apoptosis in RA-FLS (130). Therefore, ROS play a promoting role in the apoptotic process of RA-FLS. Conversely, induction of ROS production in FLS increases the level of cellular autophagy, thereby protecting FLS from apoptosis (131). Interestingly, knockdown of Atg 5 promoted the expression of RA-FLS inflammatory factors and transcriptional activity of NF-κB, which inhibited its secretion by activation of RA-FLS-specific autophagy (132). Ferroptosis is a type of cell death caused by iron-dependent lipid peroxidation (133). The mitochondrial TCA cycle and electron transport chain also promote ferroptosis progression by acting as a major source of cellular lipid peroxide production (134). Evidence has shown that ferroptosis is strongly linked to the pathological process of RA, and that induction of FLS ferroptosis helps to delay arthritis progression in CIA mice (135).

RA-FLSs have tumor cell-like anti-apoptotic characteristics, and inhibition of their proliferation and migration has been the focus of attention in RA therapy. ROS and mitochondrial damage play an important role in the proliferation and migration of FLSs, and they have emerged as important targets in RA therapy. Fang et al. (136) developed an ROS-responsive berberine polymer micelle based on the abnormal elevation of ROS in RA-FLSs, which could increase the uptake of berberine by RA-FLSs, inhibit synovial tissue proliferation, and attenuate the inflammatory response by recognizing ROS and mitochondrial superoxide. The highly effective and targeted mode of action of berberine undoubtedly provides a new direction for RA treatment.

### 3.3 Activation of inflammatory pathways

**Figure 2:**
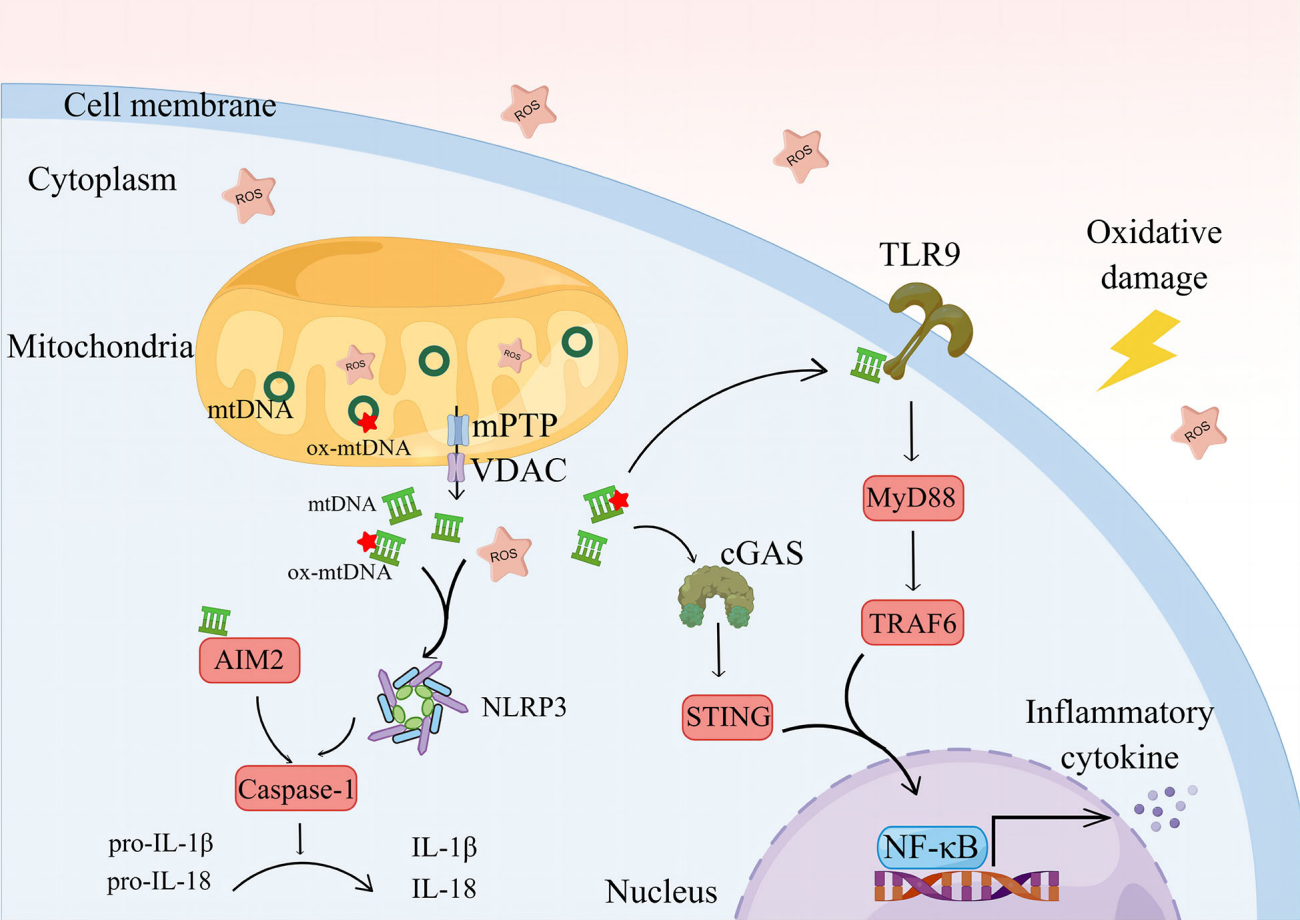
The oxidative damage environment and ROS cause mitochondrial damage, prompting mtDNA leakage and a further increase in ROS. mtDNA is oxidized by ROS to form ox-mtDNA, co-activating NLRP3 and prompted casape-1 maturation, activated IL-1β and IL-18. The cytoplasmic mtDNA can be recognized by AIM2, mediating the activation of AIM2 and promoting the cleavage of casape-1, mediating the inflammatory response. By recognizing the free mtDNA, cGAS activates the STING/NF-κB pathway in the cytoplasm.NLRP3, NOD-like receptor protein 3. IL-1β, interleukin-1β. IL-18, interleukin-18. AIM2, Absent in melanoma-2; cGAS, cyclic GMP-AMP synthase; STING, stimulator of interferon genes; TLR9, Toll-like receptors 9; MyD88, myeloid differentiation factor 88; TRAF6, TNF receptor associated factor 6; NF-κB, nuclear factor kappa-B. By Figdraw.

ROS can disrupt mitochondrial lipid membrane integrity and lead to mPTP abnormalities, resulting in oxidative damage and leakage of mtDNA (137, 138). ROS and mtDNA are PAMPs that are good activators of several inflammatory pathways and an important bridge between oxidative stress and inflammatory responses (139, 140) (**Figure 2**).

#### 3.3.1 NLRP3 inflammasome pathway

NOD-like receptor protein (NLRP)3 is a multiprotein complex that functions to activate IL-1β (141). It is associated with RA activity and inflammatory responses (142, 143). Studies have shown that tofacitinib regulates Treg/Th17 cell homeostasis by inhibiting NLRP3 inflammatory vesicle activity during RA treatment (144). ROS can promote Th17 differentiation by activating NLRP3 (145). ROS and mtDNA play a key role in the assembly of NLRP3 (146), and LPS-induced inflammatory response in macrophages involves the activation of NLRP3, a process that requires the involvement of mtROS. The removal of mtROS using molecular hydrogen (H_2_) significantly reduces NLRP3 activation and inflammatory factor production (147). The accumulation of mtROS leads to oxidative damage of mtDNA and formation of oxidized mitochondrial genes (ox-mtDNA). ox-mtDNA fragments escape into the cytoplasm *via* the mPTP and voltage-dependent anion channel (VDAC), which, in turn, initiates the assembly of NLRP3. Interestingly, mtROS do not induce VDAC oligomerization. Additionally, the escaped ox-mtDNA are recognized by cyclic GMP-AMP synthase (cGAS)/stimulator of interferon genes (STING) signaling and mediate the inflammatory response in RA. A crosstalk may occur between the cGAS/STING pathway and NLRP3 (148). In the mitochondria, the DNA glycosylase, OGG1 (mt-OGG1), can deoxidize ox-mtDNA, thereby maintaining mtDNA quality. mt-OGG1 overexpression significantly reduces ox-mtDNA content in the cytoplasm and mitochondria and inhibits NLRP3 activation (149), indicating the lack of DNA repair capacity in PBMCs of patients with RA (150). In the future, repairing specific DNA damage using clustered regularly interspaced short palindromic repeats (CRISPR) technology may produce a cure for patients with RA (151).

Additionally, NLRP3 is an important protein in mediating cell pyroptosis. Caspase-1 is a key effector protein of NLRP3. Activated caspase-1 enables the N-terminal sequence of cleaved gasdermin D (GSDMD) to bind to the cell membrane to produce membrane pores, leading to cell pyroptosis and release of a large number of inflammatory factors (152). Effectively, the GSDMD-mediated pyroptosis process promotes the release of mtDNA. Gasdermin targeted at the plasma membrane promotes mitochondrial collapse and leads to the initial accumulation of mtDNA in the cytosol (153). In a study, ROS promoted the progression of pyroptosis mediated by NLRP 3. Oxidative stress resulted in the oxidation of four amino acid residues of GSDMD in macrophages and significantly improved the cutting efficiency of caspase-1 on GSDMD (154). In RA-FLS, ROS can increase the level of caspase-1 by activating G protein-coupled receptor kinase 2 (GRK 2)/HIF-1α/NLRP 3, increase the cleavage of GSDMD, and promote the pyroptosis of FLS. Using monomeric derivatives of paeoniflorin (MDP) or removing ROS can reduce the phosphorylation of GRK 2 and inhibit FLS pyroptosis (155).

NLRP3 has now become a focus in RA research, NLRP3 inhibitors highlight the therapeutic potential. MCC950 is a small-molecule inhibitor targeting NLRP3. MCC950 intervention in CIA mice inhibited NLRP3 activation in the synovium, reduced the production of IL-1β, and alleviated joint inflammation and bone destruction (143). The JAK pathway inhibitor, tofacitinib, regulated the Treg/Th17 cell ratio in CIA mice and suppressed NLRP3 activation, whereas administration of NLRP3 abrogated this effect of tofacitinib, suggesting that NLRP3 played a pivotal role in the process of tofacitinib-mediated Th17 cell activation (144). The NLRP3 inhibitor, OLT1177, has been clinically studied in gouty arthritis and knee osteoarthritis. It has a significant effect in improving the pain of joint inflammation. However, its application is still lacking in RA (156).

#### 3.3.2 cGAS/STING pathway

The cGAS/STING pathway is mainly found in the cytoplasm. It is a key pathway mediating autoimmunity, sterile inflammation, and cellular senescence by recognizing free DNA in the cytoplasm and activating inflammatory responses (157, 158). The cGAS/STING pathway has become a hot topic in cancer research (159). TNF is a core factor mediating inflammation in RA and reduces mitophagy by inhibiting PTEN-induced putative kinase 1 (PINK1), leading to mitochondrial damage. This, in turn, increases the level of mtDNA in the cytoplasm and directly activates the cGAS/STING pathway, promoting the production of inflammatory factors. Knockdown of cGAS can significantly reduce the expression of multiple chemokines and attenuate toe joint swelling in CIA mice (160). The activation of the cGAS/STING pathway causes ROS accumulation and mitochondrial damage and promotes the migration and invasion of RA-FLSs, a process associated with the activation of the Hippo pathway. The knockdown of *FOXO1* and *MST1*, key genes of the Hippo pathway, significantly inhibits the migration and invasion of RA-FLSs (161). The cGAS/STING pathway plays a key role in the chronic inflammatory network by activating NF-κB and NLRP3 to promote the production of multiple inflammatory factors (162). Additionally, inhibition of pathway activation can play a therapeutic role in RA (163). However, its role in RA needs to be explored further.

#### 3.3.3 TLR9/NF-κB pathway

Toll-like receptor (TLR) is an early discovered class of pattern recognition receptors that play an important role in the inflammatory response to RA (164, 165). TLR9 is highly expressed in the PBMCs of patients with RA and positively correlates with the levels of inflammatory factors, such as IL-6 and TNF-α. TLR9 plays a key role in the interaction between FLSs and neutrophils (166, 167). Neutrophil extracellular traps (NETs) are a major source of guanylated autoantibodies. NET-containing guanylated peptides can be internalized by FLSs through the TLR9 pathway, elevating the inflammatory phenotype of FLSs and upregulating the expression of major histocompatibility complex class II molecules, which subsequently produce autoantibodies by presentation to Ag-specific T-cells (168). Hydroxychloroquine is a classical therapeutic agent for RA, and its therapeutic mechanism is related to the inhibition of dendritic cell (DC) activation by blocking TLR9 activation (169). The hypomethylated CpG sequence in mtDNA binds specifically to the N-terminal part of the C-shaped leucine-rich repeat region of TLR9, mediating TLR9 activation (170, 171). TLR9 activation can mediate NF-κB phosphorylation through myeloid differentiation factor 88 (MyD88) and then translate various factors, including IL-1, IL-6, and TNF-α, to mediate the inflammatory cascade (172). Additionally, studies have shown that ROS play a key role as a “secondary messenger” in regulating B-cell maturation and lgG and lgM production, which require the involvement of TLR9 (173). However, this aspect of the study has not yet been reported in RA.

#### 3.3.4 AIM2 inflammasome pathway

Absent in melanoma-2 (AIM2) inflammasome is a member of the innate immune sensor, which can detect double-stranded DNA (dsDNA), including mtDNA, in the cytoplasm independent of sequence. dsDNA can form PYD domain helical filament with AIM 2, nucleate ASC, mediate the activation of caspase-1, and induce the pyroptosis process or release active IL-1β and IL-18, which have great inflammatory potential (174). AIM2, ASC, and caspase-1 were more expressed in the knee synovium of patients with RA than those with OA. They were positively correlated with ESR and CRP levels, which may be associated with high mtDNA expression in the synovial fluid of patients with RA. Inhibition of AIM2 expression or transfection of AIM2 siRNA can significantly inhibit the proliferation and inflammatory behavior of FLS (175). ROS is a key factor leading to mtDNA leakage, and inhibition of oxidative stress contributes to reduction of mitochondrial damage, release of dsDNA, and activation of AIM2 (176). Additionally, ROS assisted in the process of AIM2 activation during bacterial infection (177).

## 4 Role of ROS and mitochondrial damage in RA angiogenesis

Pannus is a characteristic pathological product of RA and consists of neovascularization, inflammatory cells, proliferating synovial cells, and mechanized fibrin (178). Synovitis is the pathological basis for pannus. Persistent chronic synovitis leads to synovial congestion and edema and gradual accumulation of neutrophils and various immune cells in synovial tissue, whereas the proliferation of FLSs and active immune cells in synovial tissue increases the demand for oxygen and nutrient supply, forcing microangiogenesis in synovial tissue and eventually leading to a dysregulated neovascular network and the formation of villi-like proliferating granulation tissue (179–181). Therefore, the key step in the formation of pannus is angiogenesis, which provides a resupply for proliferating and migrating synovial cells and aggravates cartilage destruction and erosion (182). Mitochondrial damage in synovial tissue and oxidative stress environment are key factors in the induction of angiogenesis (38, 183). Repairing mitochondrial damage or scavenging ROS can inhibit angiogenesis (10, 184).

HIFs are major regulators that respond to ROS and mediate angiogenesis. HIFs consist of two subunits, α and β. Under normal conditions, HIFs are hydroxylated by the hydroxylase family in an oxygen-dependent manner, which leads to a substantial reduction in the transcriptional activity of HIFs, whereas under hypoxic conditions, the hydroxylate activity is inhibited, and HIFs accumulate in the cytoplasm. Activated HIFs translocate to the nucleus and rapidly transcribe various metabolic enzymes and vascular-related reactive substances to adapt to the hypoxic environment (185, 186). ROS accumulation in the RA joints prompts the high expression of HIFs, including HIF-1α and HIF-2α (35), and increase the expression of MMP-1, MMP-13, and IL-1β in FLSs. Silencing HIF-1α with siRNA significantly reduces the expression of these factors (187). HIF-1α perpetuates the interaction between synoviocytes and T and B cells, which in turn induces persistent production of inflammatory factors and autoantibodies (188). Moreover, HIF-1α can crosstalk with the TLR pathway to drive RA inflammatory response (64). Although HIF-2α shares many similarities with HIF-1α, HIF-2α and HIF-1α have been shown to differ in their sensitivities to hypoxic signaling and inflammation and can play a catabolic role in RA (189). However, both HIFs can accelerate cartilage destruction in RA (190). In addition to increasing the activity of MMPs, HIFs increase the production of chondrocyte glycolysis and the mitochondrial activity of chondrocytes under hypoxia, but ultimately leads to chondrocyte death (191). Mitochondrial ROS production in neutrophils increases the stability of HIF-1α and plays an important role in chronic inflammatory diseases (192). Nicotinamide adenine dinucleotide phosphate oxidase 4 (NOX4) increases ROS production, and stimulation of FLSs with NOX4 elevates the expression of vascular cell adhesion molecule 1 (VCAM1) and VEGF, contributing to vascular neogenesis and proliferation and migration of FLSs (193). Calreticulin (CRT) has been shown to be related to the pathogenesis of RA. CRT stimulation increases synovial NO production and phosphorylation levels of nitric oxide synthase in human umbilical vein endothelial cells (HUVECs) and promotes the proliferation, migration, and angiogenesis of HUVECs (194).

Additionally, the Notch signaling pathway responds to the regulation of ROS and has a close association with RA angiogenesis (37). In a study, Notch 1 and Notch 3 were highly expressed in RA synovial tissue. Notch 3 signaling from the vascular endothelium drove FLS activation, and mice with genetic deletion of Notch3 were resistant to serum-induced joint inflammatory responses. The Notch pathway inhibitor, LY411575, attenuated joint destruction and pannus severity in CIA rats (195, 196). Another study showed that cyclic, uniaxial stretch of human VSMCs increased Nox derived-ROS formation and Notch3 activation. Using Catalase to clear H_2_O_2_ prevented the stretch-induced translocation of Notch3 to the nucleus and decreased the Notch3 extracellular domain (197). Notch1 mediates VEGF/Ang2-induced angiogenesis and EC invasion in RA synovial tissue (198). Clearing of ROS from HUVECs inhibits Notch-induced HUVEC proliferation, migration, and adhesion (199). Besides, the Notch pathway is regulated by HIF. Notch1, Notch3 intracellular domain (N1ICD, N3ICD), and HIF-1 α were highly expressed in RASFC. Hypoxia-induced N1ICD and N3ICD expression in RASFC was blocked by siHIF-1α. Concurrently, siNotch1 and siNotch3 inhibited hypoxia-induced RASFC invasion and angiogenesis *in vitro*, whereas N1ICD and N3ICD overexpression promoted these processes (200).

ROS can transcribe VEGF through the activation of the NF-κB pathway and participate in processes, such as microvascular neogenesis and proliferation (36). In an oxidative stress environment, VEGF increases plasminogen activator (PA) and PA inhibitor-l (PAI-1) mRNA expression, increases plasminogen activator activity, hydrolyzes extracellular proteins, and thus promotes neocapillary formation (201, 202). By binding to its receptor, VEGF induces VEGF receptor phosphorylation and activates mitogen-activated protein kinase (MAPK), which induces vascular endothelial cell proliferation (203, 204). Studies have shown that VEGF gene polymorphisms are associated with RA susceptibility and activity and can be used for the clinical diagnosis and treatment of RA. High expression of VEGF can increase small vessel density in synovial inflammatory areas and elevate the levels of inflammatory factors, such as TNF-α and IL-1β (205–207).

Additionally, mitochondria play a regulatory role in angiogenesis (38). Mitochondrial thioredoxin reductase 2 (TrxR2), uncoupling protein 2 (UCP2), and panthenol-cytochrome c reductase-binding protein (UQCRB) can regulate VEGF activity and vascular endothelial activity (208). Among them, UQCRB is one of the subunits of the mitochondrial respiratory chain complex III, and mutations in UQCRB increase mtROS production and activate HIF-1 transactivation, promoting vascular neovascularization, a process that can be regulated by UQCRB inhibitors (209). FUN14 domain-containing protein 1 (FUNDC1), a protein localized on the outer mitochondrial membrane, is associated with mitophagy and mediates the formation of mitochondria-associated endoplasmic reticulum membranes, which can lead to increased cytoplasmic levels of Ca^2+^. This promotes serum response factor (SRF) phosphorylation and enhances SRF binding to the VEGFR2 promoter and leads to increased VEGFR2 transcription, leading to angiogenesis. In contrast, silencing of FUNDC1 can reverse the above process (210). Glucose-6-phosphate isomerase (GPI) is closely related to RA activity (211) and a key enzyme involved in the “Warburg effect” of RA. The accumulation of GPI is associated with abnormal mitochondrial respiratory processes (212). Hypoxic conditions can upregulate GPI activity, and in RA synovial tissue cells, upregulated GPI can induce RA angiogenesis by increasing the expression of HIF-1α and VEGF (213, 214).

## 5 Role of ROS and mitochondrial damage in RA bone destruction and cartilage damage

Articular damage is a serious complication of RA that can lead to irreversible joint deformity, severely limiting joint mobility and affecting the quality of life of patients (1). The main cause of joint damage in patients with RA is the imbalance between osteoblasts and osteoclasts, which is characterized by increased bone resorption by osteoclasts and decreased bone formation by osteoblasts, accompanied by apoptosis of chondrocytes (215, 216). Previous studies have shown that an active immune response in synovial tissue is a key factor affecting RA joint damage. Recent studies have shown that ROS and mitochondrial damage similarly modulate RA joint damage and play an important role.

### 5.1 Bone destruction

Bone destruction in RA joints presents as localized bone loss, initially involving cortical bone, disrupting the natural barrier between the external bony tissue and trabecular space of the marrow cavity. When the pannus invades the cortical bone, subchondral bone, and adjacent bone marrow cavity, eventually the trabecular bone disappears (217, 218). The tilt of RA bone metabolic balance towards bone resorption is a main factor causing bone destruction and leads to decreased bone mineral density and increased bone fragility. Therefore, patients with RA have a higher risk of fracture (219). Osteoclasts are the main players in RA bone destruction and cartilage damage. Osteoclasts are huge multinucleated cells derived from monocyte/macrophage cell lines, filling between inflammatory synovial tissue and the surface of bone joints. Through various proteases, such as cathepsin K, MMPs, and tartarate hydrochloric acid phosphatase (TRAP), they produce a local acidic environment, initiate calcium lysis, and degrade bone matrix.

Receptor activator of NF-κB ligand (RANKL) is a peptide type II transmembrane protein of the TNF superfamily that is associated with osteoclast differentiation and development, increases osteoclast bone resorption, and regulates its fate (220). Two receptors are available for RANKL; one is RANK, which is present on the cell membrane surface of osteoclast precursor cells. The binding of RANKL to RANK can promote the differentiation and maturation of osteoclasts, increase bone resorption, and delay osteoclast apoptosis. The other is osteoprotegerin (OPG), a member of the tumor necrosis factor receptor (TNFR) superfamily, with a stronger affinity to RANKL than RANK, which can competitively prevent RANKL from binding to RANK, thus inhibiting osteoclast differentiation and bone resorption activity and inducing its apoptosis. Denosumab is a monoclonal anti-RANKL antibody, which inhibits osteoclastic formation by binding to RANKL on osteoblasts. In patients with RA undergoing long-term denosumab treatment, denosumab effectively inhibited the progression of joint destruction and was generally well tolerated (221).

**Figure 3:**
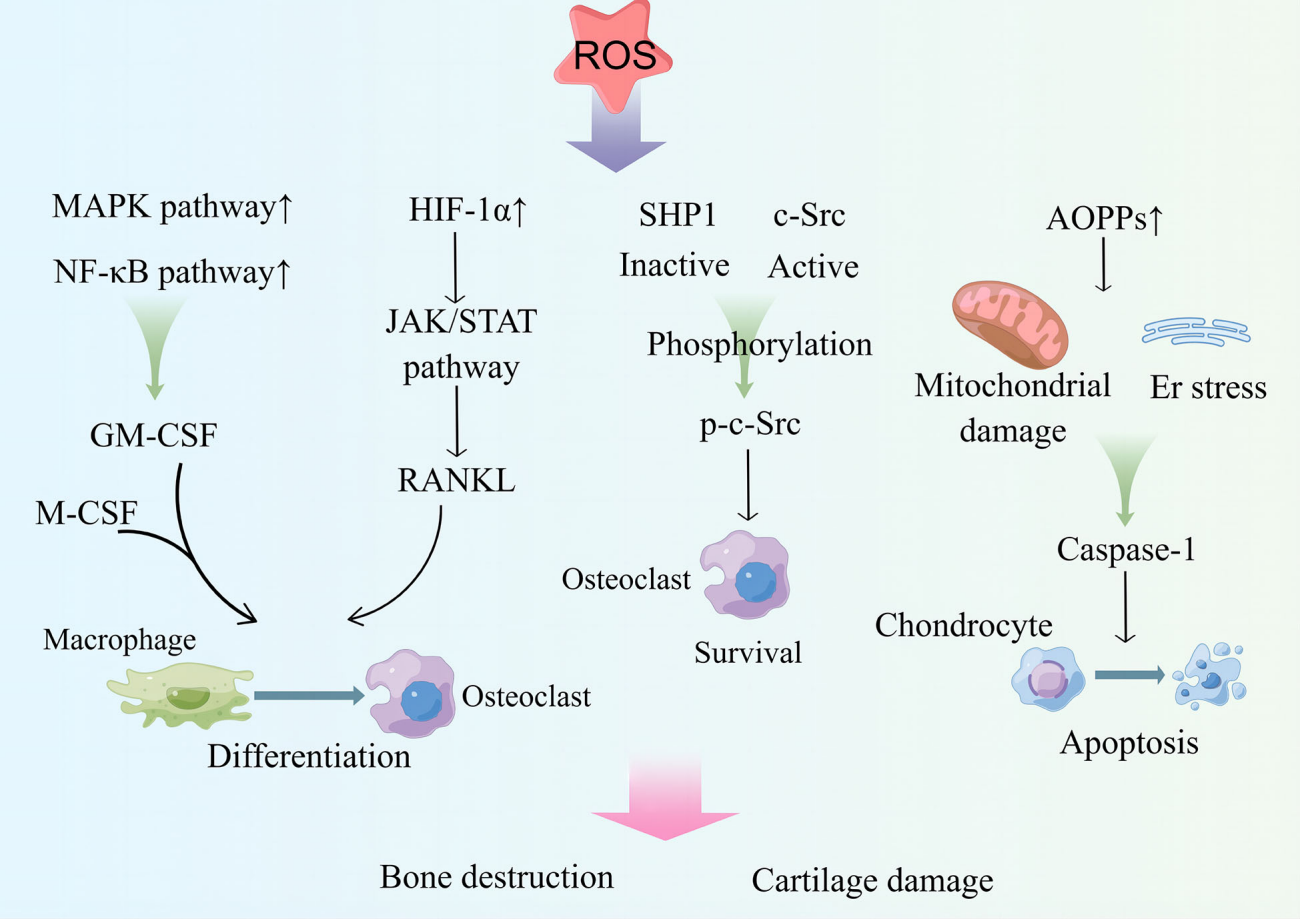
ROS upregulates MAPK and NF-κB pathway activities and transcribes GM-CSF, which acts in conjunction with M-CSF to induce osteoclast differentiation. ROS can upregulate the expression of HIF-1α, activate the JAK/STAT pathway, induce RANKL production, and promote osteoclast differentiation. ROS activate c-Src for osteoclast survival by inactivating SHP1. ROS drive the production of AOPPs, causing ER stress and mitochondrial damage; produce caspase-1; and drive chondrocyte apoptosis. These processes play an important role in bone destruction and cartilage destruction in RA. MAPK, mitogen-activated protein kinase. GM-CSF, granulocyte macrophage colony-stimulating factor. HIF-1α, hypoxia-inducible factor. JAK, Janus kinase; STAT, signal transducer and activator of transcription; RANKL, receptor activator of NF-κB ligand; SHP1, Src homology 2 domain-containing phosphatase 1; AOPPs, advanced oxidation production products. By Figdraw.

One study showed that radiation therapy in patients with malignant tumors easily leads to damage to the skeletal system. *In vitro* experiments showed that radiation can induce the ratio of ROS levels and RANKL to OPG in osteoclast precursor cells (RAW 264.7), prompting the differentiation of RAW 264.7 into osteoclasts. Intervention with the therapeutic drug, amifostine (AMI), can reduce DNA damage and ROS levels in cells and the ratio of RANKL to OPG, inhibit the maturation and differentiation of osteoclasts, and has a bone protective effect (222).Osteoclasts cause ROS accumulation during bone resorption or increased RANKL expression (223). ROS are key factors in the regulation of osteoclast differentiation (**Figure 3**). On the one hand, ROS, as a second messenger, can activate MAPK or NF-κB pathway and mediate granulocyte macrophage colony-stimulating factor (GM-CSF) production (224), which can interact with RNAKL and M-CSF to promote osteoclast differentiation (225). On the other hand, ROS induce the binding of Src homology 2 domain-containing phosphatase 1 (SHP1) to c-Src and the oxidation of c-Src and SHP-1, which lead to SHP-1 inactivation and activation of c-Src *via* phosphorylation of Tyr416, contributing to osteoclast survival and increasing bone loss (226). Additionally, ROS can upregulate HIF-1α expression, leading to activation of the Janus kinase (JAK) 2/STAT3 pathway, which promotes high RANKL expression and induces osteoclast differentiation (227). Additionally, ROS upregulation of HIF-1α can increase angiopoietin-like 4 expression in osteoclasts and enhance osteoclast activity (228, 229). Ni et al. (230) demonstrated that HIF-1α inhibits osteoclast ferritin phagocytosis and autophagocytosis in a hypoxic environment, reducing osteoclast ferroptosis. They further showed that the use of specific inhibitors of HIF-1α is effective in preventing bone loss.

Additionally, mtROS affect osteoclast differentiation. Targeted removal of mtROS using MitoQ reverses hypoxia-induced calcineurin activity and NF-κB activity and inhibits the differentiation of RAW 264.7 macrophages into osteoclasts (231). Glucose metabolism by mitochondrial oxidative phosphorylation is the main bioenergetic pathway that supports osteoclast differentiation, but increased glycolytic activity promotes osteoclast differentiation (232). The hypoxic environment of the RA joint cavity and abnormal mitochondrial respiration of FLSs lead to an increase in glycolytic activity and “Warburg effect” (70). This leads to the accumulation of lactic acid.

### 5.2 Cartilage damage

Cartilage is mainly composed of chondrocytes, outer matrix proteoglycans, and type II collagen (233). The extracellular matrix of cartilage can be degraded by MMPs. MMP-1 and MMP-13 are mainly degradable-type collagens, and non-collagen matrix protein components, such as MMP-3, are degradable proteoglycans (234). Synovial inflammation is a key driver of cartilage damage in RA, and inflammatory stimuli contribute to the high expression of multiple MMPs and RANKL in the synovial tissues of patients with RA (48). The pannus attached to the cartilage surface exacerbates inflammation and hypoxia, thereby promoting bone erosion (235). Previous studies have confirmed the above view that in the post-arthroplasty tissues of patients with RA, RNAKL is mainly expressed at the endothelial-bone interface and subchondral bone erosion sites, the site of contact between the pannus and cartilage (236). TNF-α and IL-6 promote the conversion of RANKL-induced PBMCs into osteoclasts, and PBMCs of patients with RA show a higher differentiation potential (237). Therefore, regulating the differentiation of monocytes into osteoclasts in patients with RA is an attractive target.

Cartilage destruction in RA is closely linked to the oxidative stress environment, and the hypoxic environment increases monocyte differentiation toward osteoclasts and elevates osteoclast activity (39) (**Figure 3**). ROS affect the progression of cartilage damage by regulating chondrocyte life cycle and metabolism of cartilage matrix. As a signaling intermediate, elevated ROS levels can affect growth factor bioavailability by preventing extracellular matrix (ECM) synthesis, affecting bioavailability, participating in degradation of ECM components, promoting MMP production, and inducing chondrocyte death (40). On the one hand, excessive ROS generation is involved in the process of chondrocyte growth inhibition and apoptosis promotion through signaling pathways, such as PI3K/AKT and p38 pathways (238, 239). On the other hand, ROS increases the sensitivity of chondrocytes to ROS-mediated chondrocyte death through dysregulation of GSH antioxidant system, and ROS clearance reduces chondrocyte death and enhances chondrocyte viability (41, 240).

The increased expression of advanced oxidation production products (AOPPs) in patients with RA is associated with the process of bone destruction. AOPPs can induce apoptosis in chondrocytes by triggering mitochondrial dysfunction and endoplasmic reticulum stress, leading to caspase activation, which can be blocked by the use of antioxidants (241). Furthermore, high levels of 3-nitrotyrosine in the cartilage of patients with RA induce cellular mitochondrial dysfunction and chondrocyte apoptosis through a calcium-dependent process (242). Additionally, chondrocyte death is difficult to repair, and a degree of mitochondrial autophagy is necessary for chondrocyte protection when stress occurs (243). Intervention of experimental arthritic mice with the autophagic agonist, rapamycin, reduces the severity of arthritis (244). Mitophagy is a way to repair mitochondrial damage and maintain mitochondrial homeostasis. AMPK and sirtuin (SIRT)3 are key proteins that regulate mitochondrial homeostasis. They have been shown to exert a potential protective effect on chondrocytes by maintaining mitochondrial homeostasis (245, 246). However, excessive mitochondrial autophagy may induce apoptosis in chondrocytes (247). Uncontrolled mitophagy may lead to an imbalance in cellular homeostasis and requires further investigation in RA.

## 6 Targeting ROS or mitochondria in RA therapy drugs

### 6.1 Inhibition of inflammation

Inhibition of inflammatory response is critical in the course of RA treatment, and biological agents, such as TNF-α monoclonal antibodies, IL-6 monoclonal antibodies, and JAK pathway inhibitors, have been developed for inflammatory factors and have achieved significant clinical efficacy. However, their prolonged application increases the risk of viral infection and immune suppression (248). Adalimumab is a recombinant, fully human, IgG1 monoclonal antibody. It binds specifically to TNF-α and blocks their interaction with p55 and p75 cell surface TNF receptors. It is the major drug in RA therapy (249). Some studies have explored the effects of adalimumab treatment on the global gene expression profile in PBMCs of responder patients with RA. The results showed that immune response and regulation of mitochondrial redox are the key therapeutic mechanisms of adalimumab (250). Tocilizumab is a humanized anti-IL-6 receptor monoclonal antibody. Tocilizumab with methotrexate is effective for improving the symptoms of RA in patients with inadequate response to TNFi (251). In a study on systemic juvenile idiopathic arthritis (sJIA), tocilizumab significantly altered genes regulating mitochondrial dysfunction and oxidative stress in patient neutrophils (252). Anakinra is an IL-1 receptor antagonist used for treating moderate-to-severe RA that has been unresponsive to initial disease-modifying anti-rheumatic drug (DMARD) therapy. The study showed that anakinra promotes the binding of SOD2 to the deubiquitinase, ubiquitin specific peptidase 36 (USP36), and constitutive photomorphogenesis 9 (COP9) signalosome, thus increasing SOD2 protein longevity. This effect could mediate the clearance of ROS and inhibit NLRP3 activation (253). Furthermore, in cystic fibrosis, anakinra improved the proteostatic network by coupling the mitochondrial redox balance to autophagy (254).

Therefore, exploring new therapeutic targets remains a challenge. Several studies have shown that ROS and mitochondria can be used as targets to inhibit inflammation in RA. Many active ingredients of herbal medicines have been shown to improve inflammation in RA by scavenging ROS or regulating mitochondrial function, which can provide a basis for the development of natural botanicals.

The active ingredient of leigongteng, a herbal medicine commonly used in RA treatment, has been developed as a leigongteng polyglucoside, which can be used in the clinical treatment of RA (255). Celastrol (Cel) is a quinone-methylated triterpenoid extracted from *Tripterygium wilfordii* that has been shown to alleviate inflammatory response in RA by inhibiting the ROS/NF-κB/NLRP3 axis (256). ROS-sensitive polymer micelles have been developed for Cel delivery, which can overcome the disadvantages of poor water solubility and short half-life of Cel. These micelles can alleviate RA synovial inflammation by inhibiting macrophage M1 polarization (257). Salicin from *Alangium chinense* has anti-inflammatory effects, reduces ROS production by activating Nrf2/HO-1, and inhibits inflammatory factor secretion by FLSs *in vivo* and *in vitro* (258).

Several natural drugs inhibit inflammatory responses associated with the regulation of mitochondrial homeostasis in RA. Quercetin (Que) is a major active flavonoid component isolated from *Herba taxilli*. It activates the SIRT1/peroxisome proliferator-activated receptor-gamma coactivator 1 α (PGC-1α) pathway to promote mitochondrial biogenesis, regulate mitochondrial homeostasis, and inhibit the high mobility group protein (HMGB)1/TLR4/p38/extracellular regulated protein kinases (ERK)1/2 pathway to reduce inflammatory responses in CIA mice (259). The combination of *Cornus officinalis* and *Paeonia lactiflora* was effective in ameliorating oxidative stress and inflammation in CIA rats, a process associated with the regulation of AMPK-mediated mitochondrial homeostasis. Apoptosis of synovial cells may be involved in the treatment (260). Mitochondria are closely related to the cell cycle and play key roles in apoptosis (261, 262). Many natural drugs can regulate the cell cycle and promote apoptosis in FLSs through the mitochondrial pathway (263, 264). However, unlike previous findings, shikonin, icariin, and other drugs induce mitochondrial dysfunction by increasing ROS levels, decreasing mitochondrial membrane potential, and elevating the release of cytochrome C and pro-apoptotic proteins, such as caspase-3 and caspase-9, to induce apoptosis in FLSs to suppress inflammatory response (265, 266). However, this seems to be a manifestation of drug cytotoxicity. Therefore, toxic effects should be considered when studying plant drugs.

### 6.2 Inhibition of angiogenesis

Synovial inflammatory response in RA is dependent on angiogenesis, which is mutually reinforcing and central to the progressive development of pannus. Current studies have shown that several DMARDs, such as methotrexate, can inhibit angiogenesis. Methotrexate inhibits angiogenesis in a three-dimensional co-culture model (containing synovial fibroblasts and vascular endothelial cells) and inhibits the formation of pannus (267). Leflunomide has been shown to inhibit angiogenesis-related endothelial function, and novel biologics, such as the JAK pathway inhibitors, peficitinib and tofacitinib, have been shown to treat RA by inhibiting VEGF expression and angiogenesis (268–270). The VEGF monoclonal antibody, ranibizumab, significantly improved synovial inflammation in CIA rats and was superior to the IL-6 monoclonal antibody, tocilizumab, in terms of anti-bone destruction (271). Currently, DMARDs, in combination with angiogenesis inhibitors, are considered a potential strategy for RA treatment (272). However, only a few clinical studies have reported on this treatment strategy for RA.

Abatacept (ABT) is a co-stimulation inhibitor that can bind to CD80 and CD86, preventing CD28-mediated T cell activation by blocking costimulatory signaling. In patients with RA, ABT produced significant clinical and functional benefits. Moreover, VEGF was significantly decreased in the serum of patients with RA receiving ABT (273), while transcriptomics showed that the mechanism of action of ABT is associated with improved antioxidative damage and regulation of the ETC pathway (274, 275).

Accumulation of ROS and upregulation of HIF-1α contribute to M1 cell polarization and cause inflammatory responses, whereas knockdown of HIF-1α facilitates M2 polarization (276). Kim et al. (277) developed a biocompatible therapeutic agent for ROS accumulation using manganese ferrite and ceria nanoparticle-anchored mesoporous silica nanoparticles (MFC-MSNs) joint cavity injection, which can actively clear ROS and produce O_2_. MFC-MSNs can be used as drug delivery vehicles to enhance therapeutic effects through sustained release of methotrexate (MTX). Li et al. (278) prepared ROS-responsive artesunate (ART) and dexamethasone (DEX) as a prodrug micellar nanosystem (DEX/HTA), which can effectively accumulate ART and DEX in AIA rats with arthritis. It can be specifically internalized by M1 cells, release ART and DEX, scavenge ROS, inhibit the HIF-1α/NF-κB pathway, and mediate repolarization of macrophages.

Resveratrol has been intensively investigated in several aspects of RA treatment, and it can delay the progression of RA by scavenging ROS and reducing angiogenesis by blocking the MAPK pathway (279). Liquiritin, a natural extract of *Glycyrrhiza uralensis*, has been found to inhibit RA angiogenesis. Liquiritin can promote apoptosis by regulating changes in mitochondrial membrane potential and inhibit the expression of p38 and VEGF (280). AMPK is a key protein that senses oxidative stress and regulates the body’s antioxidant activity. AMPK can improve oxidative stress by regulating SOD (SOD2) expression and mitochondrial superoxide levels and is involved in VEGF expression and angiogenesis (281). In conclusion, mitochondria are considered to be a key target for angiogenesis inhibition. However, more studies on RA are needed.

### 6.3 Inhibition of bone destruction and cartilage damage

Clinical trials have shown that the use of tocilizumab in combination with methotrexate in patients with moderate-to-severe RA can significantly decrease the level of the bone remodeling markers, C-terminal cross-linked telopeptide of type I collagen and MMP-degraded type II collagen, and inhibit the bone remodeling process (282). Therefore, early and regular drug administration is the key to reduce the disability rate of RA. A double-blind randomized controlled trial showed that treatment with the anti-RANKL antibody, denosumab, significantly inhibited the progression of joint destruction and was well tolerated, which is expected to become the clinical treatment for RA (283). Curculigoside is a glycoside in polyphenols obtained from roots and exhibit antioxidant effects. It regulates cartilage destruction, enhances osteoblast differentiation, reduces osteoclast differentiation, and inhibits osteolytic progression (284). Therefore, curculigoside has been studied extensively in bone destruction-related diseases, such as osteoporosis and RA. Network pharmacology analysis has shown that the phosphoinositide 3 kinase/protein kinase B (PI3K/AKT) pathway and proteins, such as epidermal growth factor receptor, recombinant MAPK kinase 1 (MAP2K1), and MMP-2, are key targets for curculigoside therapy (285). *In vitro* studies have shown that curculigoside inhibits tartrate-resistant acid phosphatase activity in osteoblasts induced by RANKL or H_2_O_2_ and reduces the expression of cathepsin K (Ctsk) and MMP-9. Its mechanism of action is closely related to the regulation of the Nrf2/NF-κB pathway and reduction of ROS levels (286). *Sanguis draconis* is a traditional Chinese herb, and its active ingredient, loureirin B (LrB), is widely used in the treatment of inflammatory and immune diseases. LrB can reduce RANKL-induced osteoclastogenesis by inhibiting recombinant NFAT (NFATC1) and ROS activity. It is a potential drug for the treatment of osteoporosis (287). LrB inhibits Ca^2+^ influx and IL-2 secretion in Jurkat T-cells by inhibiting the KV1.3 and stromal interaction molecule 1 (STIM1)/Orai1 pathways, which can induce immunosuppressive effects (288). It is a potential drug for the treatment of autoimmune diseases. However, it has not yet been studied in RA.

Several DMARDs have been shown to slow the progression of cartilage damage in patients with RA. Both *in vivo* and *in vitro* studies have shown that methotrexate inhibits FLS invasion and reduces cartilage degradation (289). Additionally, leflunomide treatment decreased the levels of MMP-1, MMP-9, and cartilage oligomeric matrix protein (COMP) in the serum of patients with RA (290). Meanwhile, studies have designed loaded PEI-SS-IND-MTX-MMP-9 siRNA nanoparticles for RA cartilage damage for the delivery of indindexin (IND), MTX, and MMP-9 siRNA, which significantly downregulated the expression of MMP-9 and various inflammatory factors in Raw-264.7 cells and showed anti-inflammatory activity and reversal of bone destruction in RA mice (291). Postprandial selenium supplementation can inhibit ROS and RANKL levels and reduce cartilage destruction in CIA mice. However, the optimal dose of selenium supplementation has not yet been determined, and relevant clinical studies are underway (292).

As mentioned above, multiple factors, such as ROS accumulation and mitochondria, can promote articular cartilage destruction and accelerate joint deformation in RA. Many drugs have been shown to slow the process of bone destruction by targeting ROS or mitochondrial damage. Diosmin is an unsaturated glycoside with antioxidant and anti-inflammatory properties. Intervention with diosmin and trolox (a water-soluble vitamin E that can be used to scavenge ROS) in rats administered complete freund’s adjuvant (CFA) reduced the levels of various peroxidation products and production of various MMPs and elevated the Nrf2 activity, inflammatory response, and cartilage destruction in CFA-administered rats (293). Mitochondria are equally attractive targets for stopping the process of bone destruction in RA. Estrogen levels are associated with several bone damage-phase diseases and can affect chondrocyte metabolism and cell cycle (294). Some studies have shown that 17b-estradiol (17b-E2) promotes mitophagy and enhances chondrocyte viability by elevating AMPK/mammalian target of rapamycin (mTOR) pathway activity (295). However, a cohort study showed that the role of estrogen in RA remains controversial (296). Urolithin A (UA), a natural metabolite produced by intestinal bacteria and mainly found in fruits, such as pomegranate, can improve mitochondrial function. UA reduces disease progression, cartilage degeneration, synovial inflammation, and pain symptoms in OA mouse models and may play a therapeutic role in RA, suggesting dietary advice for patients with RA (297). The Chinese herbs, turmeric, yujin, and curcumin, are commonly used in RA treatment. Their common active ingredient, curcumin, can mediate mitophagy in OA chondrocytes by promoting AMPK/PINK1/Parkin, scavenging ROS, elevating mitochondrial membrane potential, and exerting an inhibitory effect on cartilage destruction (298). Although many similarities exist between OA and RA, many differences exist because mitochondrial damage and oxidative stress are more pronounced in RA than in OA (299). However, studies on targeting mitochondria for RA treatment remain inadequate.

## 7 Summary and discussion

In recent years, studies have provided new insights into the development of RA. Oxidative stress and mitochondrial damage are inextricably linked to RA development, and our focus was on the mitochondrial regulation of metabolism affecting the disease process of RA. However, mitochondria, as a signaling center, affect RA in multiple ways. Here, we summarized the role of the vicious cycle of mitochondrial damage and ROS accumulation in RA and introduced potential drugs that target mitochondria or scavenge ROS for RA treatment, with the aim of providing some help for the clinical treatment of RA (**Table 1**).

**Table 1:** Drugs used in RA treatment that act by regulating ROS or the mitochondria.

Treatments	Mechanisms	Effects	Reference
Adalimumab	Regulation of the mitochondrial redox balance	Reduces ACPA, RF levels and decreases DAS28 scores	(250)
Abatacept	Regulation of the mitochondrial metabolism	pro-apoptotic and anti-angiogenesis	(274)
	Regulation of the mitochondrial electron transport chain	Improved joint swelling and pain, CRP levels, VAS and DAS28 scores	(275)
Tocilizumab	Improves mitochondrial dysfunction and oxidative stress	Reduced ESR, CRP levels in sJIA patients	(252)
Kurarinone	Reduces oxidative damage, inhibits Th1 and Th7 differentiation, and activates the Nrf2/HO-1 pathway	Inhibits immune inflammation	(62)
Rosmarinic acid	Induces apoptosis in activated T-cell subsets *via* the mitochondrial pathway	Inhibits immune inflammation	(76)
Resveratrol	Activates the Nrf2/HO-1 pathway, reduces ROS production, and inhibits the NF-κB pathway	Inhibits the proliferation and migration of FLSs, prompted apoptosis of FLSs	(115)
	Reduces ROS, inhibits HIF-1α and MAPK pathways, and induces FLSs G0/G1 cycle arrest	Promotes the apoptosis of synoviocytes and inhibits angiogenesis	(279)
	Regulates Nrf2/ARE activity and reduces ROS *via* the SIRT1/NF-κB/miR-29a-3p/Keap1 and SIRT1/NF-κB/miR-23a-3p/cul3 axis	Inhibits the proliferation of FLSs and reduces oxidative damage	(114)
	Inhibits autophagy in FLSs, decreases mitochondrial membrane potential, and increases mtROS level	Promotes apoptosis of FLSs	(131)
Celastrol	Inhibits the ROS/NF-κB/NLRP3 axis system	Inhibits the inflammatory response	(256)
	Regulates the NF-κB and Notch1 pathways and suppresses M1 polarization in response to ROS	Reduces synovial inflammation	(257)
Salicin	Inhibits the Nrf2/HO-1/ROS pathway	Inhibits the viability of FLSs and reduces oxidative damage	(258)
Quercetin	Promotes mitochondrial biogenesis by regulating the SIRT1/PGC-1α/NRF1/TFAM pathway, alleviating the inflammatory response, suppressing HMGB1/TLR4/p38/ERK1/2/NF-κB p65	Restrains synovial inflammation and cartilage destruction	(259)
*Cornus officinalis* and *Paeonia lactiflora* Pall.	Regulate mitochondrial dynamics *via* AMPK and inhibit ROS production	Reduce inflammatory cytokines and promote apoptosis in synovial tissues	(260)
Icariin	Depresses the mitochondrial membrane potential, increases ROS production, and induces a G2/M phase arrest in FLSs	Inhibits the proliferation and migration of FLSs and promotes apoptosis of FLSs	(265)
Shikonin	Activates ROS, mediates mitochondrial damage, and activates the PI3K/AKT/mTOR pathway	Induces autophagy and apoptosis in FLSs	(266)
MFC-MSNs	Eliminate ROS, produce O_2_, regulate the HIF-1α pathway, and induce M1 to M2 polarization	Inhibit synovial inflammation	(277)
DEX/HTA	Eliminates ROS, induces M1 to M2 repolarization, and inhibits the HIF-1α/NF-κB pathway	Reduces synovial inflammatory infiltration and repairs articular cartilage injury	(278)
Liquiritin	Regulates ΔΨm and inhibits JAK, p38, and VEGF expression	Inhibits FLS proliferation and promotes apoptosis and angiogenesis	(280)
Diosmin and trolox	Reduce the serum levels of iNOS, NF-κB, and MMPs and activate the Nrf2 pathway	Anti-inflammatory and anti-oxidative stress activities and relieve bone erosion	(293)
Curculigoside	Inhibits the ROS, NOX1, NOX4, and NF-κB pathway activity and increases the Nrf2 activity	Reduces oxidative stress and inhibits osteoclast formation	(286)
Loureirin B	Inhibits ROS and MAPK/NFAT pathway activity and decreases Ctsk and Atp6v0d2 expression	Inhibits osteoclast formation and reduces bone resorption	(287)
Metformin	Induces mitophagy by enhancing the membrane potential of the SIRT3/PINK1/Parkin pathway	Regulates the ECM balance of chondrocytes and reduces cartilage destruction	(300)
Curcumin	Induces mitophagy *via* activation of the AMPK/PINK1/Parkin pathway and eliminates ROS	Promotes chondrocyte survival and reduces cartilage destruction	(298)

Although progress has been made in the study of mitochondria in RA, many questions remain unanswered. Mitochondrial damage induces mitochondrial division, which in turn promotes mitochondrial autophagic behavior to remove damaged mitochondria and induce mitochondrial regeneration. However, different studies have reported different results, with some showing that inhibition of dynamin 1-like protein expression and mitochondrial division can inhibit mitophagy and alleviate inflammatory response in RA (301). However, some studies have reported contrasting results (260). This may be related to the diversity of mitochondrial division forms and great variations in the outcomes caused by different division forms (302). Additionally, the roles of ROS and mitophagy in RA are complex and diverse, with ROS inducing mitochondrial autophagic behavior and enhancing mitochondrial autophagic activity, possibly contributing to the survival of FLSs and increasing the inflammatory response, and inhibition of autophagy inducing apoptosis of FLSs (303). Elevation of ROS levels to promote apoptosis has been widely studied in cancer research (304). Therefore, the two-fold nature of mitophagy and its role in RA need to be studied further (305).

We have summarized many potential therapeutic agents for mitochondrial damage and ROS accumulation. Although ROS or mitochondria is a reliable target for RA treatment, many therapeutic agents that are being used in the clinics can play a role in repairing mitochondrial damage or scavenging ROS. However, the advantages and differences in these potential therapeutic agents, compared with existing drugs, require further research and exploration. Much work is needed before potential drugs can be used in clinical treatment.

## Author contributions

WJ wrote the manuscript, CL proofread the manuscript. XD and HW revised the manuscript. All authors contributed to the article and approved the submitted version.
